# Comparison of Laboratory and Field Remote Sensing Methods to Measure Forage Quality

**DOI:** 10.3390/ijerph7093513

**Published:** 2010-09-27

**Authors:** Xulin Guo, John F. Wilmshurst, Zhaoqin Li

**Affiliations:** 1 Department of Geography, University of Saskatchewan, 117 Science Place, Saskatoon, Saskatchewan, S7N 5C8, Canada; E-Mail: zhl237@mail.usask.ca; 2 Jasper National Park of Canada, Parks Canada, P.O. Box 10, Jasper, AB T0E 1E0, Canada; E-Mail: John.Wilmshurst@pc.gc.ca

**Keywords:** forage quality, chemical contents, remote sensing, mixed-grass prairie, protein, NDF, ADF

## Abstract

Recent research in range ecology has emphasized the importance of forage quality as a key indicator of rangeland condition. However, we lack tools to evaluate forage quality at scales appropriate for management. Using canopy reflectance data to measure forage quality has been conducted at both laboratory and field levels separately, but little work has been conducted to evaluate these methods simultaneously. The objective of this study is to find a reliable way of assessing grassland quality through measuring forage chemistry with reflectance. We studied a mixed grass ecosystem in Grasslands National Park of Canada and surrounding pastures, located in southern Saskatchewan. Spectral reflectance was collected at both *in-situ* field level and in the laboratory. Vegetation samples were collected at each site, sorted into the green grass portion, and then sent to a chemical company for measuring forage quality variables, including protein, lignin, ash, moisture at 135 °C, Neutral Detergent Fiber (NDF), Acid Detergent Fiber (ADF), Total Digestible, Digestible Energy, Net Energy for Lactation, Net Energy for Maintenance, and Net Energy for Gain. Reflectance data were processed with the first derivative transformation and continuum removal method. Correlation analysis was conducted on spectral and forage quality variables. A regression model was further built to investigate the possibility of using canopy spectral measurements to predict the grassland quality. Results indicated that field level prediction of protein of mixed grass species was possible (r^2^ = 0.63). However, the relationship between canopy reflectance and the other forage quality variables was not strong.

## 1. Introduction

Recent research in rangeland ecology has emphasized the importance of forage quality as a key indicator of rangeland condition [1–4]. Given the opportunity, grazing animals select forage of high nutritional quality, which usually means that they are selecting forage that is not the most abundant [5–9]. Forage quality has been frequently reported to affect the behavior of mammalian herbivores (e.g., [10–12]). However, the evaluation or mapping of forage quality at temporal and spatial scales appropriate for animal management is a challenge, although it can improve understanding of animal behaviour.

Forage quality can be expressed *via* grass chemical composition and nutrient concentration. Chemical composition mainly refers to protein, lignin, ash, moisture (at 135 °C), Neutral Detergent Fiber (NDF), Acid Detergent Fiber (ADF), and Total Digestible, which directly influences food particle digestion by grazing animals [13]. Nutrients mainly mean Digestible Energy (DE), Net Energy for Lactation (NEL), Net Energy for Maintenance (NEM), and Net Energy for Gain (NEG), which can also influence the production of animals [14]. Considering the importance of on the health and production of herbivores, a great number of efforts have been made on evaluating forage quality. The traditional approaches usually were implemented requiring detailed sampling and expensive laboratory analyses, which are time-consuming, tedious, pricy, and most importantly, less representative of the population in large areas [15].

Superior to the traditional methods, the application of remote sensing makes it possible to evaluate and predict forage quality of rangeland timely and efficiently, especially in large areas [16]. Estimation of forage chemical composition via a remote sensing approach can be dated back to late 1970s [17–19]. However, the main remote sensing approach, namely the near infrared spectroscopy (NIRS, the typical analyzed wavelength range is 1,100–2,500 nm), can only provide accurate biochemical measures of protein, amino acids, lignin and cellulose concentrations in dry foliage in laboratory [20–22]. Extending the NIRS approach to a canopy level in the field has yielded limited success, largely because of the masking effects of water in fresh canopies [23–26]. Recently, hyperspectral remote sensing technique has been applied to evaluate forage quality in the field [27,28]. Starks *et al.* [29] compared the estimation of NDF and ADF from the approaches of laboratory chemical analyses, NIRS, and close range hyperspectral remote sensing, and found that accurate estimation of forage composition can be obtained through the hyperspectral data in warm season pasture land in Oklahoma, USA. The hyperspectral data were also successfully used to predict the biochemicals of living vegetation in tropical savanna rangeland in South Africa [16,30–32]. In addition, the research conducted in a sown pasture land in Hokkaido, Japan also suggested that the pasture quality (protein, ADF, NDF) can be predicted by *in situ* canopy hyperspectral reflectance [33]. However, a big concern about the application of hyperspectral remote sensing remains due to the fact that *in situ* canopy reflectance may be heavily influenced by atmospheric variation [34], soil background and leaf orientation and distribution [35]. Such a concern may be even bigger in northern semi-arid mixed grasslands, which are characterized by a large amount of bare soil and dead material [36,37]. Despite the concern, few studies have compared the estimation of forage chemical composition using *in situ* hyperspectral canopy reflectance measured in the field and *ex situ* hyperspectral reflectance data measured for dried grass in the laboratory. In addition, the application of hyperspectral remote sensing is also influenced by the mathematical methods used to establish the relationship between canopy reflectance and forage quality [23,33], for example, Mutanga *et al.* [16] found the continuum absorption approach is better than band width on predicting forage chemical composition.

Grazing could affect the nutritive value of the forage [38,39] and the effects would change as the grazing density change, which were concluded from an experiment in a moist grassland in Czech Republic [40] and a southern mixed grass prairie in the USA [41]. But little research has been focused on the effects of light to moderate grazing on forage quality in northern semi-arid mixed grass prairie. Therefore, the objectives of our study are three-fold: (1) to evaluate hyperspectral measurements for estimating the forage chemical composition of northern semi-arid mixed grass prairie in the field and laboratory, (2) to test the reliability of a field level grass quality prediction method, and (3) to compare the effects of grazing on grassland quality estimation.

## 2. Materials and Methods

### 2.1. Study Sites

The study was conducted in Grasslands National Park of Canada (GNP) (West Block) and surrounding pastures owned by the federal and provincial government and private ranchers in southern Saskatchewan, Canada. Dominant vegetation in this community includes needle-and-thread (*Hesperostipa comata Trin. & Rupr.*), blue grama (*Bouteloua gracilis (HBK*) *Lang. ex Steud*.) and western wheatgrass (*Pascopyrum smithii Rydb.*). Spikemoss (*Selaginella densa Beauv*.) and Junegrass (*Koeleria macrantha (Ledeb*) *J.A. Schultes f*.) are also frequently observed [42]. Characterized by the semi-arid mixed prairie ecosystem, this region receives approximately 340 mm annual precipitation primarily falling in the growing season (May–September). The mean annual temperature is 3.4 °C with the maximum mean daily temperature of 20 °C in July and the minimum of −15 °C in January [43]. Variation in grassland quality due to long-term changes in productivity or elevated atmospheric CO_2_ are of a concern to land managers in this region.

### 2.2. Data Collection

Data were collected in the field and laboratory. Fieldwork was conducted in June–July of 2003. Thirty sites were randomly selected within upland grasslands of the study area. At each site, two 100-m transects perpendicular to each other oriented in the cardinal directions were surveyed. Field level canopy reflectance was collected at 5 m intervals along each transect (40 readings at each site) using an ASD FR Pro spectroradiometer (Analytical Spectral Devices, Inc., USA) to capture within-site variation. The wavelength measurement range was 350–2,500 nm, and the spectral resolution was 3 nm at 700 nm and 10 nm at 1,400 and 2,100 nm. A 25° field of view probe was used pointing down to the canopy at approximately 1 m above ground, yielding a view area of the surface of about 0.15 m^2^. Measurements were taken between 1,000 h and 1,400 h local time under cloud-free conditions. Calibration was made using a white reflectance panel (Labsphere, USA) at approximately 10 minute intervals to minimize solar variation due to the changing sun angle over the measurement period. Reflectance (R) in a given waveband was calculated by dividing the canopy spectra by the white reference spectra. After the canopy reflectance was measured, above-ground vegetation was clipped within a 20 × 50 cm quadrat at 20 m intervals along each transect, which yields 12 clipped samples at each site. Vegetation samples were sorted into green grass, forb, shrub, and dead material shortly after clipping and then dried in an oven at 65 °C for 24 hours. Dry green grass samples randomly selected from 360 samples collected on 30 sites were cut into 1 cm lengths and spread out to 1 cm thickness on a black surface for measuring indoor spectral reflectance [44]. Indoor spectral reflectance was then measured in the laboratory, using the ASD Pro Lamp for illumination, which is specifically designed for indoor lab reflectance measurements over the region of 350–2,500 nm. Therefore, spectral measurements were made on *in situ* field-canopy samples and *ex situ* dry green grass in laboratory, and used for developing models for evaluating forage quality. After the indoor spectral measurements, the dry samples were then sent for chemical analysis (ETL ChemSpec Analytical Services Ltd.).

### 2.3. Spectral Data Processing

Hyperspectral remote sensing provides detailed information at small wavelength intervals. But this also generates a great volume of data that can be difficult to interpret. Interpretation is aided by processing the data using algorithms that aggregate regions with known information value. The most commonly used hyperspectral data processing methods are the first derivative transformation and continuum-removed absorption algorithm. **First derivative reflectance:** frequently, the clearest patterns in reflectance data occur not in the absolute quantity of reflectance, but rather the rate of change of reflectance from one wavelength to another. The first derivative transformation of the reflectance spectrum (R_fd_), that calculates the slope values from the reflectance, can be derived from the following equation [45]:

(1) $$R_{f\;d(i)}=(R_{\left(j+1\right)}-R_{\left(j\right)})/\Delta_{\lambda }$$

where *R_f d_* is the first derivative reflectance at a wavelength *i* midpoint between wavebands *j* and *j* + 1. *R*_(*j*+1)_ is the reflectance at wavelength *j* + 1, and Δ_λ_ is the difference in wavelengths between *j* and *j* + 1.

**Continuum removal:** the continuum removal algorithm proposed by Kokaly and Clark [46] is a popular method for absorption feature detection. Spectral absorption regions are selective for chemical composition and density in vegetation because higher concentrations of particular chemicals lower the reflectance in unique spectral regions. Six known chemical absorption regions were selected: two (carotenoids and anthocyanins) in the visible range that are related to chlorophyll absorption and nitrogen concentration (R_470–518_ and R_550–750_), and four in the shortwave infrared region (R_1116–1284_, R_1634–1786_, R_2006–2196_, and R_2222–2378_), that are the result of lignin, protein and other chemical absorption [16,26,47]. For the six defined absorption regions, a linear continuum was identified from the start to the end points. Differences between the measured value and the continuum were calculated and then summed to represent accumulated absorption in the given region. The other approaches for hyperspectral data processing, such as band depth, band depth ratio, and normalized band depth index, were not tested in this study. The reason is that the research of Mutanga *et al.* [16] indicated the continuum-removed derivative reflectance (CRDR) is superior to them for estimating forage chemical composition.

### 2.4. Data Analysis

Descriptive statistics [mean, standard deviation (Std), minimum (Min), maximum (Max), and coefficient of variation (CV)] of forage chemical variables and nutrient contents were calculated to understand the general forage quality in the study area. To identify the best indicators for estimating chemical components from laboratory and field conditions, Pearson’s correlation analysis was conducted between chemical composition and the raw reflectance of dry samples and canopy reflectance, and the first derivative reflectance as well as the accumulated absorption calculated from the continuum removal approach. The relationships were plotted against wavelength regions for comparisons.

With one-fifth observed protein data and the accumulated absorption data (six each) set aside for validation, the stepwise regression analysis was applied to the other four-fifth data (24) to develop a model for predicting protein using field measured accumulated absorption derived from reflectance. The developed regression model was validated using the jackknife and cross validation approach. This approach operates by withholding the spectral data for one site and building the model functions using the data from the remaining sites. The process of removing one site from the dataset was repeated until all sites had been withheld [48]. Finally, the performance of the prediction model was evaluated by root mean-squared error (RMSE) and average relative error (ARE) by comparing the predicted values to the six observed protein values.

The differences in both chemical components and spectral absorption features between grazed and ungrazed grasslands were investigated through the analysis of variance (ANOVA), to document the effects of grazing on the detection of grassland forage quality.

## 3. Results and Discussion

### 3.1. Result

#### 3.1.1. Vegetation chemical and nutrient contents

Results of the laboratory chemical analysis of the forage quality variables of interest are summarized in Table 1. Most forage quality variables showed a small CV, indicating a relative homogeneity among samples. The only two exceptions are protein and ash content. The relatively high CV of the protein content among samples indicates the diversity of protein content of grass at the field level. Although nutritional requirements of ruminant herbivores vary with physiological state and with body size [49], benchmark values do exist. For a 500 kg mean live weight cow, the requirement of protein content is 3.1% for maintenance and 10.7% for producing 30 kg milk/day. The protein content in the study area is at above the cow maintenance level but is lower than the milk production level.

#### 3.1.2. Comparison of reflectance measured in the field and laboratory on detecting grass chemicals

**Figure 1.**
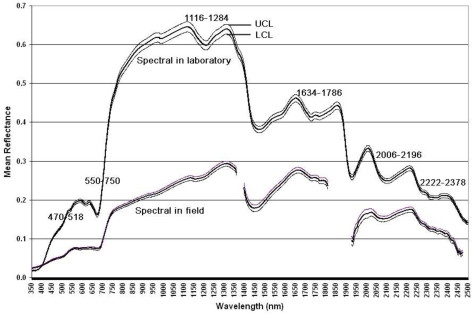
Spectral response curves of dried grass in the laboratory and living vegetation (canopy reflectance) in the field in Canadian mixed grass prairie with 95% upper and lower confident limits (UCL & LCL) of 30 samples. Noisy regions due to water vapor absorption (1,361–1,395 nm, 1,811–1,925 nm, 2,475–2,500 nm) were deleted for the field measurements.

**Spectral features:** Figure 1 illustrates the spectral features of the samples in both laboratory and field measures. The upper and lower 95% confident limits based on the 30 samples indicated that the spectral variation is small, which indicates relatively little variation over the sampled areas. Six absorption regions selected for this study were also clearly identifiable. Generally, field level spectral measures were lower than laboratory measures, which is likely due to the influence of bare ground and dead materials. The leaf orientation in the field may also contribute to the difference in the spectral response curve from the measures of dried grass samples in the laboratory which minimize the effects of leaf orientation and distribution.

**Figure 2.**
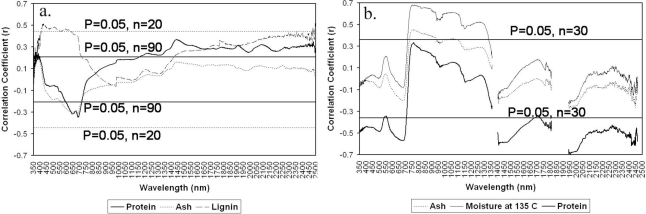
Relationships between chemical components and spectral reflectance measured in both laboratory and field. The horizontal lines are the critical significant r values for (a) 90 samples (protein and ash) and 20 samples (lignin) in the laboratory, and (b) 30 samples in the field.

**Relationships between grass quality variables and raw reflectance:** Correlation analysis on chemical composition and raw reflectance showed that only lignin, protein and ash were significantly related to reflectance of dried grass in the lab (Figure 2a) whereas protein, moisture and ash were significantly related to canopy reflectance in the field (Figure 2b).

The visible wavelength region is important: the red region (600–700 nm) correlates with protein and ash, and the blue (400–500 nm) and green regions (500–600 nm) are correlated with lignin concentration. The important red-edge region for estimating protein is consistent with the finding of Kawamura *et al.* [33] in a sown pasture land in Japan and of Starks *et al.* [50] in a warm season pasture land in USA, but the blue region (415–460 nm) was also well correlated with protein content in their study. In addition, we found no significant relationship between chemical contents and spectral features beyond the near infrared energy range. This finding agrees with [51] indicating that the visible wavelength region is the most important for grassland biophysical characterization. However, accumulated absorption regions showed significant relationships with the spectral measurements for lignin and protein, indicating a potential of using the continuum removal approach for prediction.

**Figure 3.**
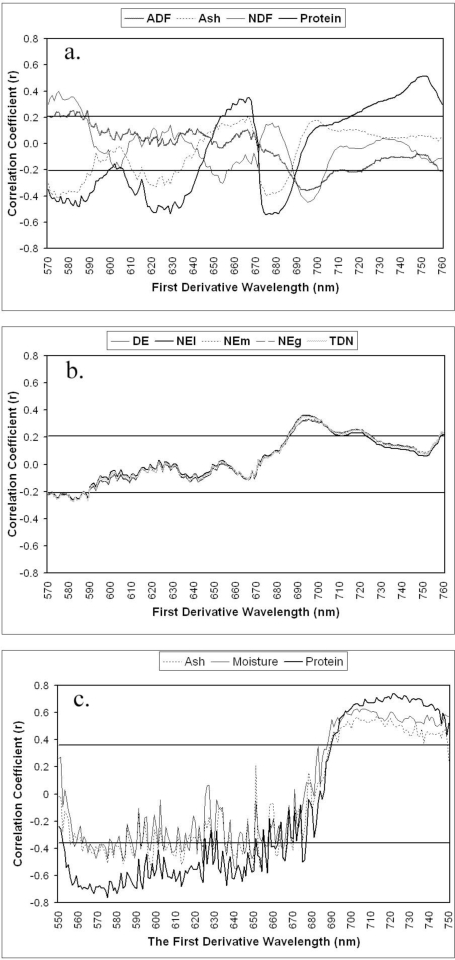
Comparisons of relationships between chemical contents and the first derivative reflectance collected in both (a, b) laboratory and (c) field.

**Relationships between quality and first derivative reflectance:** By definition, higher first derivative reflectance will occur at steeply sloped regions of the spectral response curve (Figure 1). The most obvious region is at the red edge, popularly used for Nitrogen content estimation of crops and grasses [52,53]. The first derivative of a subset of the red edge region (550–750 nm), showed a dramatic enhancement of the relationship between forage quality variables and spectral measures (Figure 3a). Energy variables showed very similar results, due to similar calculation methods (Figure 3b). The significant regions in field level measurements are in the blue and red wavelength ranges (Figure 3c).

We also compared the effects of the first derivative transformation (550–750 nm) applied to lab and field spectral measurements. In general, the first derivative transformation increased our ability to estimate chemical composition. The relationships between chemical composition and the first derivative reflectance are much more consistent based on spectral reflectance measurements in the lab throughout the wavelength region, even though the relationships were higher for the field measures for most chemical variables. The only exception is lignin content, which is more highly correlated to lab-measured reflectance than that in the field.

**Relationships between chemicals and the areas of absorption:** For most foliage chemicals, the mid infrared region is important for qualitative detection, but not for quantitative estimation. Considering the limitations of the first derivative method, the continuum removal method was tested. Accumulated absorption was correlated with forage quality variables (Table 2).

All relationships between the forage quality variables and the 470–518 nm, 550–750 nm, 1,116–1,284 nm, and 1,634–1,786 nm wavelength regions were statistically significant. Despite significant correlation, the variation of forage quality variables accounted for by the variation of spectral reflectance varied from 5–66%, indicated by the r^2^ values. Interestingly, based on the spectral measurements in the lab, the two mid wavelength regions (2,006–2,196 nm and 2,222–2,378 nm) did not show significant correlations with any chemical components. Nonetheless, based on the canopy reflectance in the field, the wavelength region (2,006–2,196 nm) demonstrates moderate to high correlation with protein, ash, and moisture, and the region of 2,222–2,378 nm is moderately correlated with protein content. In the lab, ash and moisture content only weakly correlated with the 1,116–1,284 nm wavelength region, but in the field both ash and moisture are moderately correlated with two visible wavelength regions, one near-infrared and one mid-infrared wavelength region. In addition, NDF was only correlated with 470–518 nm wavelength region, but other chemical variables correlated with more than one spectral absorption region in the lab.

#### 3.1.3. Protein prediction model development and validation

**Figure 4.**
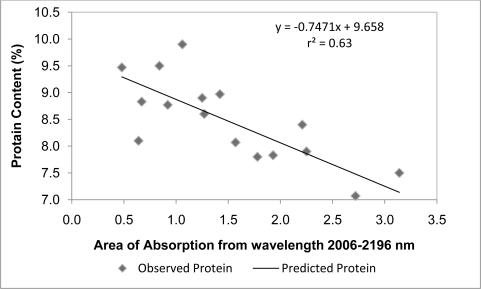
Jackknife cross validation of protein prediction model based on the accumulated absorption of field measured spectra.

When using the six absorption areas derived from field spectral measurements as independent variables in a stepwise regression analysis, we could estimate 63% variation of protein content from the absorption area of the wavelength region of 2,006–2,196 nm with the following equation:

(2) $$\text{Protein}=9.658-0.747\;\Sigma \;A_{R(2006–2196)}\;(\text{N}=24,\;{\text{r}}^{2}=0.63,\;\text{P}=0.000)$$

where A represents accumulated absorption area in the wavelength range indicated in the equation; N is the sample number; and P is the significance value at the 0.05 significance level. This suggests that the model is very good at monitoring protein from *in situ* field samples (Figure 4). Higher protein content results in smaller absorption areas due to its strong energy absorption capability in these particular wavelength regions. Therefore, larger absorption areas have smaller protein content.

Six observed protein data and the corresponding accumulated absorption areas were set aside and used to evaluate the performance of the prediction model. RMSE and ARE between the observed and predicted protein values were shown in Table 3. RMSE indicates that the predicted protein data series is quite consistent with the observed protein. ARE shows the overall error of the model prediction is 8%, which further indicates that the model (Equation (2)) can accurately predict protein based on the *in situ* measures of canopy reflectance.

#### 3.1.4. Variation under different management practices

Forage quality variables and the reflectance of the wavelength regions used for calculating the accumulated absorption in grazed and ungrazed grassland were compared in Table 4. ANOVA analysis indicated that canopy reflectance of all the selected wavelength regions is significantly different at the 0.05 significance level in grazed and ungrazed grasslands. Except lignin and protein, the other tested chemical composition, including ADF, Ash, moisture at 135 °C, and NDF, is also significantly different. As for the ADF, ash, moisture, NDF, and Lignin, both the range and CV were higher in grazed sites than those in the ungrazed area, indicating a higher variation in grazed swards. However, both range and CV indicate that the variation of protein is smaller in grazed sward than that in ungrazed sward. In addition, variation of reflectance in the wavelength regions of 1,116–1,284 nm and 1,634–1,786 nm is smaller, but that in other wavelength regions is larger, in grazed sward than ungrazed sward, indicated by CV. With biomass as a covariate, we only found that NDF (F1, 87 = 12.3, P < 0.001) and ADF (F1, 87 = 3.5, P = 0.055) differed significantly among grazing treatments and no quality measure varied significantly with biomass. In both cases, NDF and ADF were higher under the ungrazed than those under the grazed treatments (Table 4).

### 3.2. Discussion

Our data show that the strongest correlation between forage quality and remote sensing data was not from *ex situ* spectral reflectance measurements of dried grass samples in the laboratory, but from *in situ* reflectance measures made on the vegetation canopy. Previous research has indicated that water could mask the relationship in fresh samples [25,26]. However, our reflectance values from *in situ* samples showed consistently higher correlations to chemical composition than from dried samples, despite the field samples being a mixture of green grass, forb, shrub, dead materials, and soil.

Relationships between vegetation quality and spectral measurements differed when measured under laboratory conditions compared to field conditions for a range of chemical variables. Noise was less for indoor spectral measurement, but correlations to chemical composition were also lower. Both the first derivative transformation and absorption continuum removal methods worked very well to improve the relationships between chemical and spectral data. However, each method has its own suitable wavelength regions; the first derivative transformation was for absorption and reflectance transition regions especially the red edge area, while the absorption removal method was mainly for absorption regions that can be extended to mid infrared regions. Furthermore, the first derivative transformation provided more promising results for measurements in a laboratory, which provided smoother spectral response curves. Sixty-six percent variation of protein content could be explained by the total absorption area in the wavelength region of 2,006–2,196 nm. Lignin showed a better relationship with indoor spectral data than with the field measurements. The reason for this result is not fully understood as the sample numbers are lower for lignin compared to other chemical variables because of the high cost for lignin laboratory analysis. Further analysis with increased sample numbers should be investigated. Both first derivative and continuous removal approaches are specifically for hyperspectral remotely sensed data. Fortunately, data from hyperspectral satellite sensors are available currently (e.g., Hyperion on Earth Observing-1 and CHRIS on Proba). Other satellite-based hyperspectral sensors such as Canadian HERO will be launched in the future. At the same time, airborne hyperspectral imagers (e.g., AISA Airborne Hyperspectral Imager of UPM-APSB and HYMAP of Australia) also become available. However, the practical application of hyperspectral remote sensing will not be applicable in the short term due to high cost.

To verify if the remote sensing data at field level could be used for grass quality assessment, we made detailed comparisons on field and dried samples. Except lignin content, all field spectral measurements provided a better indication of chemical composition than spectral measures in the lab. The trend among wavelength regions was clear: higher negative correlations in absorption regions and higher positive correlations in reflectance regions for protein, with significantly positive correlations in near infrared region for moisture content and ash. For the lab measures, only the visible region showed a significant positive relationship with lignin content, which has no significant relationship with field spectral measurements. To measure forage quality using spectral reflectance in such a ecosystem, apparently it is not necessary to dry samples. This is consistent with the findings of Starks *et al.* [50], Mutanga *et al.* [16], Mutanga and Skidmore [30–32], and Kawamura *et al.* [33] for forage quality estimation in warm season pasture land in Oklahoma, USA, tropical savanna rangeland in South Africa, and in a sown pasture land in Hokkaido, Japan, respectively.

The interplay between plant moisture, nutritional quality, grazing and reflectance is interesting, which deserves further exploration. It appears that in more mesic vegetation communities, maturational variation in plant quality will be greater, but our ability to detect these changes using *in situ* reflectance will be greatly impaired. Plant maturation is the factor that largely dictates nutritional quality [54]. Nutritional quality typically declines as maturation increases [55]. Water stress, for its part, typically retards maturation in plants [56]. On average, nutritional quality in plant under water stress will be higher than those in well watered plants [57]. Thus, the rate at which nutritional quality declines with maturation, typically indicated by biomass and growing season, will slow. Unfortunately, in plant canopies containing more moisture, nutritional quality estimated by canopy reflectance is less reliable [16,21,29,58]. So where nutritional quality of swards likely varies as environmental conditions change, our ability to track the variation of nutritional quality will be constrained.

Additionally, forage quality is improved by short-term grazing pressure due likely to the delay of average maturity of plant tissues caused by grazing in the sward [59]. Grazing can also affect reflectance signals by creating more bare ground and reducing the density of non-photosynthetically active material in a canopy [60]. Again, grazing creates a more temporally and spatially dynamic vegetation canopy that, because of what changes are made, make forage quality detection using reflectance measures more difficult. Hence, where moisture and grazing are the dominant effects on vegetation quality, in *situ* measures of nutritional quality of forage will be challenging. This supposition is consistent with our findings. In the semi-arid grassland, variation in nutritional quality was very low and was only related to grazing treatment (not biomass). Hence our *in situ* reflectance measures were as effective as or more effective at detecting plant chemical composition than *ex situ* measures. But these factors also point to why the literature is divided on the effectiveness of *in situ* measures of forage quality. We should perhaps not expect to find similar results on more mesic grasslands or where grazing is more intense.

This conclusion has interesting implications for tracking large-scale changes in forage quality resulting from increasing atmospheric CO_2_ concentrations and climate change. As CO_2_ concentrations increase, plant growth rates are expected to increase [61] and communities may shift towards greater dominance by C_3_ species [3,62–65]. In many ecosystems these changes may be accompanied by changing precipitation patterns that will result in longer periods of little or no precipitation during the growing season [66,67]. Because C_3_ species tend to be of higher nutritional quality than C_4_ species [68] and decline less in quality with maturation [69], a shift towards C_3_ species accompanied by less growing season precipitation suggests less within-season variation in forage quality in temperate grassland ecosystems. Hence, while lower canopy moisture will enhance *in situ* detection of canopy quality variation that may accompany climate change, there may be less within-season variation to detect.

Landscape level measurement of grassland nutrient content shows a great promise with modern remote sensing tools. Our study supports the idea that grassland quality assessment using remote sensing approaches can be successful in the field. Nevertheless significant challenges, such as teasing out sward moisture effects and accounting for grazing management, remain. The key to overcoming these challenges, we believe, will be the careful comparison of controlled grazing treatments in grassland ecosystems differing in precipitation regimes.

## Figures and Tables

**Table 1.** Chemical variables derived from sample analysis (N is the sample number).

**Chemicals**	**Abbr.**	**Unit**	**N**	**Mean**	**Std**	**Min**	**Max**	**CV**
Lignin	Ln	%	20	4.03	0.33	3.47	4.54	0.082
Protein	Pt	%	90	8.41	1.02	6.2	12	0.121
Ash	Ash	%	90	6.35	1.06	4.3	11	0.167
Moisture at 135 °C	Mt	%	90	6.13	0.23	5.7	6.8	0.038
Neutral Detergent Fiber	NDF	%	90	65.47	2.11	57	70.7	0.032
Acid Detergent Fiber	ADF	%	90	35.49	1.5	30.1	39.4	0.042
Total Digestible Nutrients	TDN	%	90	54.67	1.58	50.69	59.83	0.029
Digestible Energy	DE	Mcal/kg	90	2.39	0.07	2.2	2.63	0.029
Net Energy for Lactation	NEI	Mcal/kg	90	1.23	0.04	1.13	1.36	0.033
Net Energy for Maintenance	NEm	Mcal/kg	90	1.16	0.05	1.01	0.49	0.043
Net Energy for Gain	NEg	Mcal/kg	90	0.63	0.05	0.49	0.8	0.079

**Table 2.** Correlation coefficient (r) between chemical components and accumulation of absorption features.

**Variables**	**Methods**	**Width ranges (nm)**					
		**470–518**	**550–750**	**1,116–1,284**	**1,634–1,786**	**2,006–2,196**	**2,222–2,378**
Protein	Lab	\	.328**	\	.213*	\	\
	Field	.641**	.695**	.645**	−.508**	−.812**	−.567**
Ash	Lab	\		−.267*	\	\	\
	Field	.425*	.484**	.388*	\	−.483**	\
Moisture	Lab	\		−.231*	\	\	\
	Field	.489**	.547**	.503**	\	−.445*	\
ADF	Lab	−.429**	−.250*	−.258*	−.237*	\	\
NDF	Lab	−.218*	\	\	\	\	\
TDN	Lab	.465**	.278**	.297**	.254*	\	\
DE	Lab	.463**	.280**	.291**	.256*	\	\
NEL	Lab	.461**	.263*	.300**	.246*	\	\
NEm	Lab	.455**	.279**	.288**	.253*	\	\
NEg	Lab	.456**	.273**	.282**	.241*	\	\

** Correlation is significant at the 0.01 level;

* Correlation is significant at the 0.05 level; and “\” means no significant relationship.

**Table 3.** The performance of the protein prediction model evaluated by root mean-squared error (RMSE) and average relative error (ARE).

**Absorption area**	**Observed Protein**	**Predicted Protein**	**RMSE**	**ARE**
0.5	10	9.28	0.63	0.08
0.79	8.7	9.07		
1.31	8.97	8.68		
1.98	7.53	8.18		
2.65	7	7.68		
2.09	7.2	8.10		

**Table 4.** Comparisons of chemical composition and canopy reflectance under grazed and ungrazed conditions (N is the sample number; and Mt and Ln stands for moisture at 135 °C and Lignin, respectively).

**Category**	**Statistics**	**Chemical variables**					**Protein**	**Wavelength regions**					
		**ADF**	**Ash**	**Mt**	**NDF**	**Ln**		**470–518**	**550–750**	**1,116–1,284**	**1,634–1,786**	**2,006–2,196**	**2,222–2,378**
	N	45	45	45	45	10	45	15	15	15	15	15	15
	Mean	35.2	6.6	6.2	64.7	3.9	8.3	0.06	0.10	0.28	0.27	0.17	0.14
	Std.	1.51	1.29	0.25	2.14	0.38	0.92	0.01	0.01	0.01	0.02	0.02	0.01
**Grazed**	Min	30.1	4.3	5.7	57	3.47	6.2	0.05	0.08	0.25	0.24	0.14	0.11
	Max	37.5	11	6.8	68.4	4.54	10.2	0.06	0.11	0.30	0.29	0.19	0.16
	Range	7.4	6.7	1.1	11.4	1.07	4	0.02	0.02	0.05	0.06	0.06	0.05
	CV	0.04	0.2	0.04	0.03	0.1	0.11	0.09	0.07	0.05	0.06	0.10	0.09
	N	45	45	45	45	10	45	15	15	15	15	15	15
	Mean	35.8	6.1	6	66.3	4.1	8.5	0.05	0.09	0.25	0.25	0.15	0.13
	Std.	1.43	0.73	0.15	1.77	0.24	1.12	0.01	0.01	0.01	0.02	0.02	0.01
**ungrazed**	Min	32.4	4.3	5.7	61.8	3.8	6.8	0.04	0.08	0.24	0.23	0.13	0.11
	Max	39.4	7.7	6.4	70.7	4.54	12	0.06	0.10	0.28	0.28	0.18	0.15
	Range	7	3.4	0.7	8.9	0.74	5.2	0.02	0.02	0.05	0.05	0.06	0.04
	CV	0.04	0.12	0.03	0.03	0.06	0.13	0.12	0.07	0.04	0.06	0.12	0.11
	Sig.	0.03	0.05	0.00	0.00	0.13	0.36	0.01	0.00	0.00	0.00	0.01	0.00
